# Association between weight-adjusted-waist index and periodontitis risk: A cross-sectional study

**DOI:** 10.1371/journal.pone.0302137

**Published:** 2024-05-16

**Authors:** Xinyu Wu

**Affiliations:** School of Stomatology, Jinan University, Guangzhou, China; Hamadan University of Medical Sciences, School of Public Health, ISLAMIC REPUBLIC OF IRAN

## Abstract

**Background:**

There may be an association between obesity and periodontitis, yet no studies have investigated the correlation between the new obesity indicator, the weight-adjusted-waist Index (WWI), and periodontitis.

**Objective:**

This study aims to investigate the association between the novel obesity index, weight-adjusted-waist index, and periodontitis.

**Subjects and methods:**

WWI was utilized to assess obesity, through measuring waist circumference (WC) and body weight. We analyzed cross-sectional NHANES data from 2009 to 2014 (1) using multivariate logistic regression to explore WWI’s association with moderate/severe periodontitis; (2) conducting subgroup analyses and interaction tests; and (3) fitting smoothed curves to the age-stratified logistic regression model.

**Results:**

The study involved 11,256 individuals, with 48.55% having moderate/severe periodontitis. Upon adjusting for all relevant variables, a significant correlation between WWI and moderate/severe periodontitis was observed (OR = 1.08, 95% CI: 1.01–1.17). Compared to the lowest quartile of WWI, there was a significant increase in the likelihood of moderate/severe periodontitis in Quartile 2 (OR = 1.21, 95% CI: 1.06–1.39) and Quartile 3 (OR = 1.23, 95% CI: 1.07–1.42). Subgroup analyses for gender, age, education, smoking, and diabetes highlighted a positive association between WWI and moderate/severe periodontitis in all subgroups, except for the diabetic population and individuals aged 65 years and older.

**Conclusion:**

The analysis revealed a positive correlation between WWI, a novel obesity index, and moderate/severe periodontitis prevalence through diverse modeling approaches.

## Introduction

Periodontitis is a persistent inflammatory ailment affecting the gingiva and supporting structures of the dentition. It is a multifactorial, intricate, and deleterious condition in which oral microorganisms serve as the instigating factor. The ailment is a primary contributor to tooth mobility and resorption of the alveolar bone, and in severe instances, it may culminate in tooth loss [1]. In addition to its direct impact on oral health, periodontitis is intricately linked to heightened susceptibility to systemic conditions such as diabetes, cardiovascular illnesses, cancer, inflammatory responses, and psychological stress [2]. Factors including obesity, diabetes, malnutrition, and an increased risk of periodontitis have been linked with sedentary behavior [3, 4].

Obesity is characterized by abnormal adipose tissue accumulation and represents a complex and chronic inflammatory disease and metabolic disorder, as well as a significant global health challenge [5]. An epidemiological study projected that by 2030, roughly 50% of adults in the United States are going to be obese [6]. While body mass index (BMI) is a normal parametric for gauging overweight, it fails to differentiate between fat and muscle distribution [7]. The weight-adjusted waist index (WWI) is becoming more important as people become more recognized for the risks associated with visceral fat. This index normalizes waist circumference (WC) by body weight and better assesses abdominal obesity and metabolic disturbances. WWI emphasizes the benefits of waist circumference and is considered superior to BMI and WC in evaluating muscle and adipose tissue mass [8]. Extensive studies have expounded a positive relation between WWI and the occurrence of newly diagnosed hypertension, diabetes, and overall as well as cardiovascular mortality [9, 10]. WWI may indicate age-related alterations in adipose and muscle tissue composition applicable to all population groups and may more accurately predict the risk of chronic diseases [11].

Research has found a link between overnutrition and activation of innate immunity in organs, collectively known as adiposity-related inflammation [12]. Obesity is considered one of the systemic diseases/metabolic disturbances that affect periodontal attachment and is often accompanied by metabolic disturbances and chronic inflammation [13]. BMI is widely used for obesity assessment, however, its correlation with periodontitis is still debated [14, 15]. The connection between obesity and periodontitis could be due to the limitations of traditional obesity assessment tools like BMI and WC in evaluating fat distribution. The link between obesity and periodontitis may involve insulin resistance due to inflammation and oxidative stress, but further research is needed to understand the underlying mechanisms [16]. The relationship association between obesity and periodontitis is a recent area of focus in periodontal research, and there is a lack of investigation into the association between WWI and periodontitis.

As such, this investigation intends to explore the connection amidst WWI and the incidence of periodontitis utilizing data from the National Health and Nutrition Examination Survey (NHANES), comprising a cohort of over 11,000 individuals. The goal is to investigate whether changes in WWI are related to periodontitis and whether the assessment of abdominal obesity using the easily measured WWI can help evaluate health risks associated with periodontitis.

## Materials and methods

### Data source

The National Health and Nutrition Examination Survey (NHANES), conducted by the National Center for Health Statistics (NCHS) at the Centers for Disease Control and Prevention (CDC), collects nationally representative data on diet, laboratory measurements, physical exams, interviews, and demographics using a multistage, stratified, probability sampling design. Informed consent is obtained from all participants prior to data collection. The data collection has been approved by the Institutional Review Board of the National Center for Health Statistics, and the files are made publicly available online [17]. NHANES provides a comprehensive description of the data collection procedures and methodologies [18]. The NHANES protocol was approved by the National Center for Health Statistics (NCHS) Research Ethics Review Board, and informed consent was obtained from all participants. Informed consent was obtained from all study participants. Written consent has been obtained from the participants for the publication of this paper. The NCHS Research Ethics Review Board approved the NHANES study protocols (Continuation of Protocol #2011–17; Protocol #2011–17; Continuation of Protocol #2005–06). The data that support the findings of this study are publicly available in the National Health and Nutrition Examination Survey for data users and researchers throughout the world. (www.cdc.gov/nchs/nhanes/).

### Study population

The numbers utilized in this investigation were derived from NHANES, a thorough cross-sectional survey administered by the National Center for Health Statistics (NCHS) for the assessment of the well-being and dietary situation of the U.S. populace. NHANES utilizes a rigorous layered multi-stage probability selection technique, guaranteeing a characteristic sample [19, 20].

**Fig 1:**
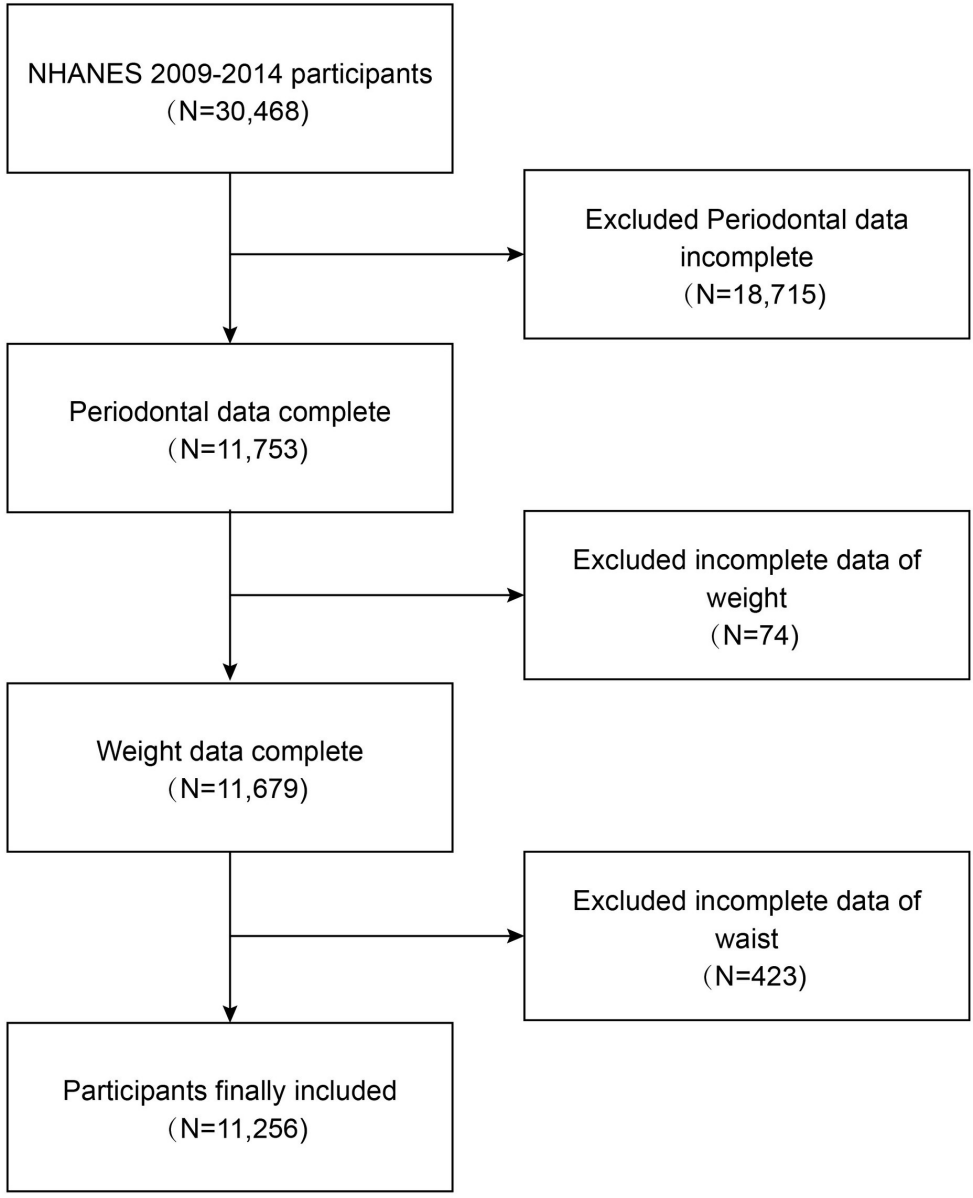
Flowchart of the sample selection from NHANES 2009–2014. A total of 30,468 participants were enrolled at first, and after the exclusion of participants, periodontal data was incomplete (n = 18,715), missing the data about weight (n = 74), waist (n = 423), 11,256 eligible participants were included in our final analysis. NHANES, National Health and Nutrition Examination Survey.

As depicted in Fig 1, our study explicitly utilizes data from three NHANES survey cycles from 2009 to 2014. A total of 11,256 subjects were incorporated into our ultimate analyses subsequent to excluding individuals without complete periodontal examination data (n = 18,715), those with missing weight data (n = 74), and individuals with missing waist circumference data (n = 423).

### Evaluation of the weight-adjusted-waist index

The WWI serves as an anthropometric indicator employed for assessing levels of obesity through measurements of waist circumference (WC) and weight. An elevated WWI score denotes an increased degree of obesity. Skilled health technicians gathered body measurements in the MEC (Mobile Examination Center). To compute the WWI for each participant, the waist circumference in centimeters was divided by the square root of the weight in kilograms, rounded to the nearest hundredth.

For this study, WWI was considered an uninterrupted factor for this investigation. Individuals were subsequently categorized into groups based on WWI quartiles for further examination. In this study, WWI was explicitly selected and utilized as the exposure variable.

### Evaluation of periodontal examination

All participants in this study underwent comprehensive examinations conducted by proficient dentists. The precise examination procedures were detailed in the operation manuals accessible on the NHANES website. We utilized the 2012 CDC/American Academy of Periodontology (AAP) case definition of periodontitis to categorize periodontal disease. Based on previous studies, to standardize the periodontal examination criteria, only participants aged 30 and above were included in this study [21].

In this study, mild periodontitis was characterized as having two interproximal sites with a clinical attachment loss (CAL) of 3 mm, along with two proximal sites with a periodontal pocket depth (PPD) of 4 mm (not on the same tooth), or one proximal site with a PPD of 5 mm.

To address the potential bias arising from the high prevalence of mild periodontitis in the population, we designated moderate/severe periodontitis as the primary outcome variable [22]. For this purpose, we defined moderate periodontitis as having two CAL 4-mm proximal sites (not on the same tooth) or two PPD 5-mm proximal sites (not on the same tooth). On the other hand, severe periodontitis was recognized by the presence of two interproximal sites with a clinical attachment loss of 6 mm (not on the same tooth) and at least one interproximal site with a periodontal pocket depth of 5 mm.

These definitions were specifically formulated to ensure consistency and minimize bias during assessing periodontitis severity.

### Covariables

Covariates included age (in years), sex (male/female), high-density lipoprotein cholesterol (HDL-C, mg/dL), race (Mexican American/Other Hispanic/Non-Hispanic White/Non-Hispanic Black/Other Races), diabetes status (Yes/No), household income to poverty ratio (PIR), education level (Less than high school/High school/More than high school), smoking status (Yes/No), and alcohol use (in days). In terms of smoking, individuals who had previously smoked 100 cigarettes or more were classified as smokers., while those who had smoked less than 100 cigarettes were considered non-smokers. Alcohol consumption was recorded based on the quantity of days the individual consumed alcohol in the previous 12 months. For additional information on variable collection methods, please refer to the NHANES Survey Methodology and Analysis Guide. All detailed measurement protocols for these variables are publicly available at www.cdc.gov/nchs/nhanes/.

### Statistical analysis

All statistical procedures were performed following the recommendations provided by the Centers for Disease Control and Prevention (CDC). Continuous variables were expressed as means and standard deviations, while categorical variables were expressed as percentages. In order to assess the demographic characteristics of participants in WWI quartiles, t-tests (for continuous variables) and chi-square tests (for categorical variables) were employed. Multivariate logistic regression models were used for investigating the independent association between WWI and moderate/severe periodontitis across three different models. WWI was transformed from a continuous variable to a categorical variable (quartiles), and a trend test was applied to examine the linear relationship between WWI and moderate/severe periodontitis. Subgroup analyses were conducted using stratified multivariate logistic regression models, which are akin to the fully adjusted model in the multivariate logistic regression analysis. The adjustment includes covariates other than the grouping factors themselves and is categorized by sex, age (≥30 and <65 years/≥65 years), smoking status, and diabetes. Additionally, smoothed curve fitting and a generalized additive model (GAM) were used for assessing the potential nonlinear association between WWI and moderate/severe periodontitis, combined with the results of stratified multivariate logistic regression modeling for age stratification. A p-value lesser than 0.05 was considered statistically significant. All analyses were conducted utilizing Empower software (version 4.1) and R (version 4.2).

## Results

### Baseline characteristics of participants

Table 1 displays the baseline characteristics of our study individuals. The study comprised 11,256 individuals, with a mean age of 53.11 ± 14.58 years. Gender distribution was 49.71% male and 50.29% female. WWI quartiles were as follows: Quartile 1: 8.62–10.59, Quartile 2: 10.59–11.11, Quartile 3: 11.11–11.65, Quartile 4: 11.65–14.79. Approximately 48.55% of participants had moderate/severe periodontitis, and its prevalence increased with higher WWI quartiles (Quartile 1: 41.83%, Quartile 2: 48.40%, Quartile 3: 51.99%, Quartile 4: 51.99%, p < 0.001). Significant differences across WWI quartiles were noted in age, gender, race, education, alcohol consumption, PIR, and HDL-C levels (p < 0.001). Smoking status did not show statistically significant differences (p = 0.192). Individuals in the highest WWI quartile had a higher likelihood of being female, Non-Hispanic White, and older than those in the bottom quartile. Higher WWI scores were associated with lower educational attainment and income levels, elevated HDL cholesterol levels, and a higher prevalence of diabetes. Among the WWI range of 10.59–11.11, the highest frequency of alcohol consumption was observed.

**Table 1:** Baseline characteristics of the study population according to WWI quartiles.

Variables	Q1	Q2	Q3	Q4	*p*-value
	N = 2814	N = 2814	N = 2814	N = 2814	
**Age, (years)**	45.39±12.04	51.06 ± 13.50	55.53 ± 14.01	60.47 ± 14.24	<0.001
**Periodontitis, (%)**					<0.001
Yes	41.83	48.40	51.99	51.99	
No	58.17	51.60	48.01	48.01	
**Sex, (%)**					<0.001
male	60.87	55.26	48.22	34.47	
female	39.13	44.74	51.78	65.53	
**Race, (%)**					<0.001
Mexican American	7.14	13.61	16.45	17.45	
Other Hispanic	7.07	9.52	12.19	11.23	
Non-Hispanic White	44.46	42.96	41.58	46.16	
Non-Hispanic Black	27.83	20.15	19.33	16.03	
Other Races	13.50	13.75	10.45	9.13	
**Education level, (%)**					<0.001
Less than high school	14.75	23.70	27.51	35.18	
High school	21.39	20.22	22.81	22.92	
More than high school	63.86	56.08	49.68	41.90	
**Smoking, (%)**					0.192
Yes	44.60	45.59	46.98	47.05	
No	55.40	54.41	53.02	52.95	
**Diabetes, (%)**					<0.001
Yes	4.05	8.39	15.64	25.69	
No	95.95	91.61	84.36	74.31	
**PIR**	2.93 ± 1.70	2.70 ± 1.65	2.54 ± 1.63	2.17 ± 1.50	<0.001
**Drinking alcohol, (day)**	5.65 ± 31.21	6.37 ± 49.19	4.89 ± 31.36	4.48 ± 41.56	<0.001
**HDL-C (mg/dL)**	57.22 ± 17.42	52.88 ± 16.27	50.66 ± 15.04	49.96 ± 14.18	<0.001

Note: p < 0.05 indicates statistical significance. In the case of continuous variables, this is obtained using the Kruskal Wallis rank sum test, and in the case of counting variables with a theoretical number < 10, this is obtained using the Fisher exact probability test.

Abbreviations: PIR, household income to poverty ratio; HDL, high-density lipoprotein cholesterol; WWI, weight-adjusted-waist index.

### A higher weight-adjusted-waist index is linked to a higher probability of moderate/severe periodontitis

The findings of this study revealed a positive association between WWI and the incidence of moderate/severe periodontitis (Model 1: OR = 1.23, 95% CI: 1.17–1.29; Model 2: OR = 1.15, 95% CI: 1.08–1.21). This association remained consistent even after thoroughly adjusting the fully adjusted model (Model 3) (OR = 1.08, 95% CI: 1.01–1.17). Consequently, each increases of 1 unit in WWI was associated with an 8% increased prevalence of moderate/severe periodontitis (Table 2). To assess sensitivity, WWI was converted from a continuous variable to categorical quartiles. Accounting for key demographic factors, the incidence of moderate/severe periodontitis exhibited an upward trend with higher WWI values. Notably, the highest WWI quartile showed an 11% amplified risk compared to the lowest quartile (Quartile 1) (OR = 1.26, 95% CI: 1.11–1.42, P for trend = 0.0002). Furthermore, Quartile 2 displayed a significant 21% increased probability, while Quartile 3 showed a notable 23% increment compared to the lowest WWI quartile. However, the disparity between Quartile 4 and Quartile 1 did not achieve statistical significance (OR = 1.14, 95% CI: 0.98–1.34) (Table 2).

**Table 2:** Association between WWI and prevalence of moderate/severe periodontitis.

Exposure		Model 1 [OR (95% CI)]	Model 2 [OR (95% CI)]	Model 3 [OR (95% CI)]
Weight-adjusted-waist index		1.23 (1.17, 1.29)	1.15 (1.08, 1.21)	1.08 (1.01, 1.17)
**Weight-adjusted-waist index (quartile)**				
Quartile 1	8.62–10.59	reference	reference	reference
Quartile 2	10.59–11.11	1.30 (1.17, 1.45)	1.17 (1.05, 1.31)	1.21 (1.06, 1.39)
Quartile 3	11.11–11.65	1.51 (1.36, 1.67)	1.26 (1.12, 1.41)	1.23 (1.07, 1.42)
Quartile 4	11.65–14.79	1.51 (1.36, 1.67)	1.26 (1.11, 1.42)	1.14 (0.98, 1.34)
P for trend		<0.0001	0.0002	0.1068

Note: Model I—unadjusted. Model II—adjusted for age, sex, and race. Model III—Model II additionally adjusted for high-density lipoprotein cholesterol (HDL-C, mg/dL), diabetes status, household income to poverty ratio (PIR), education level, smoking status, and alcohol use. p < 0.05 indicates statistical significance.

Abbreviations: OR, odds ratio; WWI, weight-adjusted-waist index.

**Fig 2:**
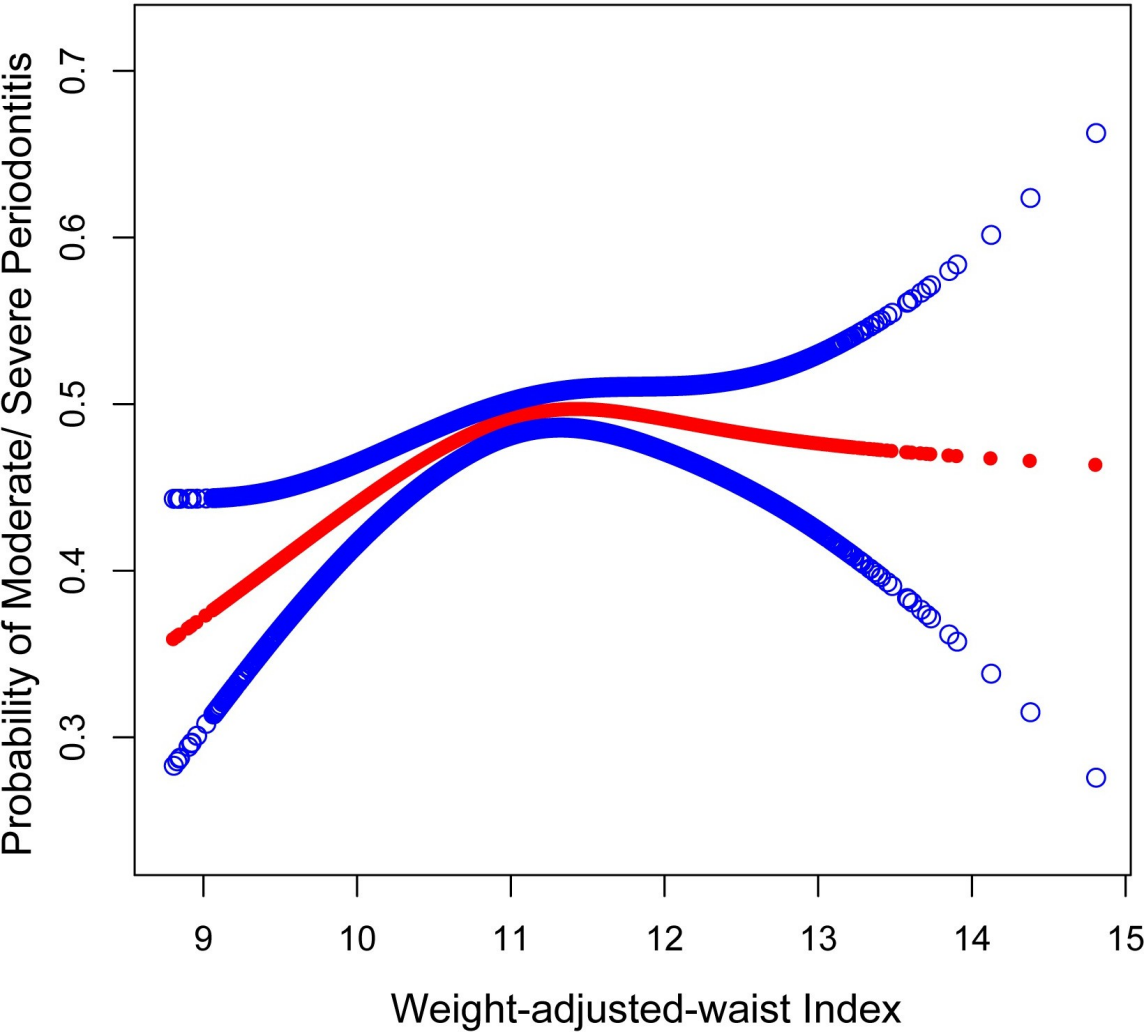
Smooth curve fitting for WWI and probability of moderate/severe periodontitis. A positive relationship between WWI and the probability of moderate/severe periodontitis was detected by the generalized additive model. WWI, weight-adjusted-waist index.

Employing smoothed curve fitting and generalized additive models (GAM), this study investigated the potentially positive relationship between WWI and moderate/severe periodontitis. The results showed a positive concurrence between these variables (Fig 2).

### Subgroup analysis

**Fig 3:**
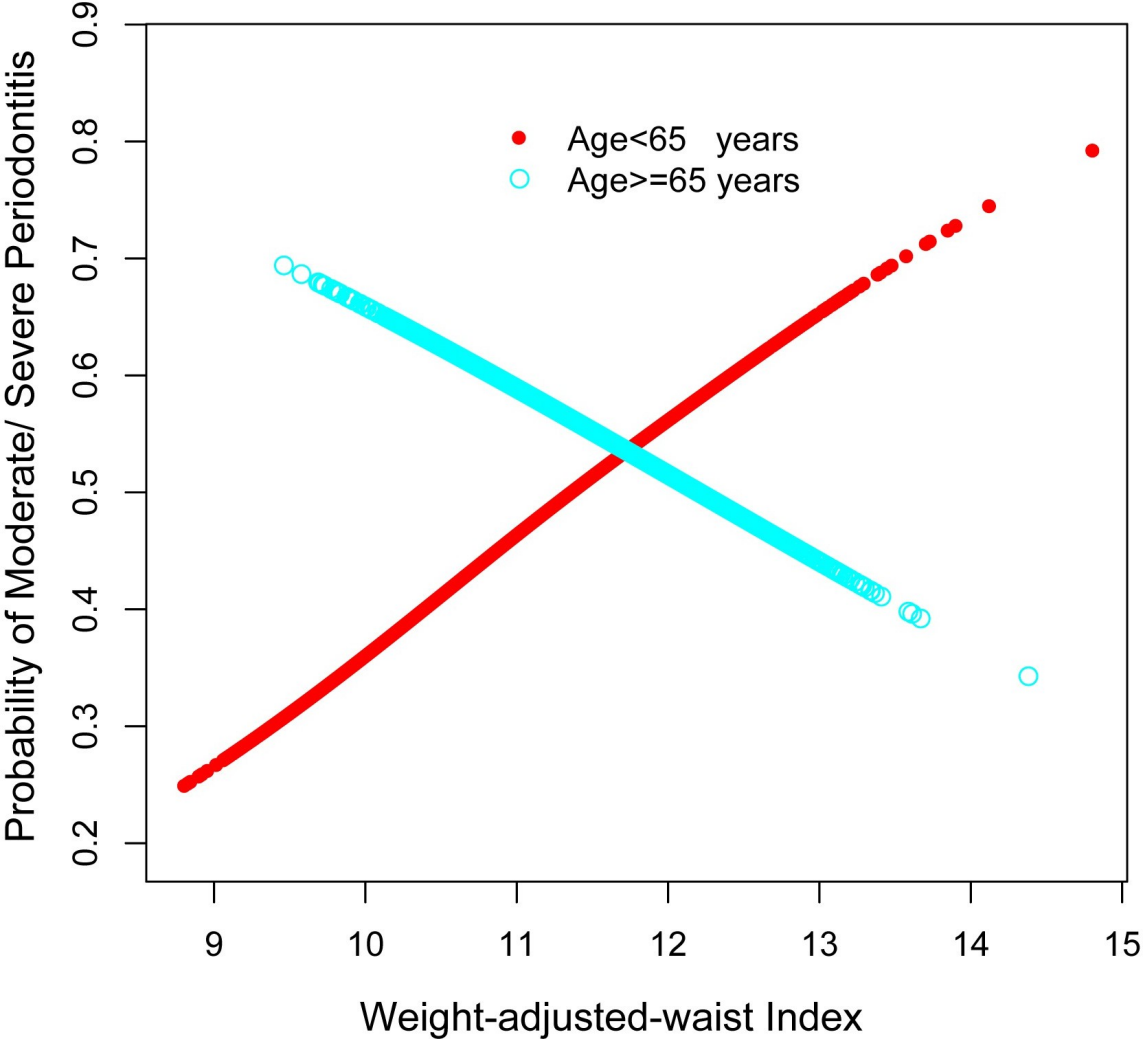
The association between WWI and moderate/severe periodontitis differed significantly between the elderly group (≥65 years) and the middle-aged group (≥30 and <65 years). WWI, weight-adjusted-waist index.

Subgroup analyses were conducted to provide insight into the potential impact on the relationship between WWI and moderate/severe periodontitis in different populations. Gender, age, education, smoking status, and diabetes mellitus were considered variables (Table 3). Age modified the association between WWI and the likelihood of moderate/severe periodontitis (P for interaction < 0.001). Among those under 65, an increase in WWI was significantly associated with increased odds of moderate/severe periodontitis (OR = 1.49, 95% CI: 1.37–1.61). In contrast, among those aged 65 years and older, an increase in WWI was associated with a slight decrease in the likelihood of moderate/severe periodontitis (OR = 0.79, 95% CI: 0.69–0.92) (Fig 3). Favorable associations between WWI and moderate/severe periodontitis were observed in most subgroups, except for diabetics and individuals aged 65 years and over.

**Table 3:** Stratified multivariate logistic regression models of moderate/severe periodontitis.

Moderate/ Severe Periodontitis	OR (95% CI)	P for interaction
**Sex**		0.8011
Male	1.08 (0.97, 1.20)	
Female	1.10 (0.99, 1.22)	
**Age**		<0.0001
<65 years	1.49 (1.37, 1.61)	
≥65 years	0.79 (0.69, 0.92)	
**Education level**		0.9248
Less than high school	1.09 (0.93, 1.27)	
High school	1.06 (0.91, 1.24)	
More than high school	1.10 (0.99, 1.22)	
**Smoking**		0.3630
Yes	1.13 (1.02, 1.25)	
No	1.05 (0.94, 1.18)	
**Diabetes**		0.2869
Yes	0.97 (0.80, 1.19)	
No	1.10 (1.01, 1.19)	

Note: Age, sex, race, high-density lipoprotein cholesterol (HDL-C, mg/dL), diabetes status, household income to poverty ratio (PIR), education level, smoking status, and alcohol use were adjusted. p < 0.05 indicates statistical significance.

### The positive interdependency association between weight-adjusted-waist index and the likelihood of moderate/severe periodontitis in a group of young and middle-aged adults

There existed a positive correlation between WWI and the incidence of moderate/severe periodontitis when segmented by gender, education level, smoking status, and diabetes mellitus. The resultant age curves exhibited significant associations among participants in the elderly group (≥65 years, N = 2,759) and the middle-aged group (≥30 and <65 years, N = 8,497). Nevertheless, the middle-aged group displayed dissimilar significant associations. Specifically, WWI showed an inverse correlation with the likelihood of moderate/severe periodontitis in the elderly age group (≥65 years) and a positive correlation in the young and middle-aged group (≥30 and <65 years) (Fig 3).

## Discussion

In this cross-sectional study encompassing 11,256 participants aged 30 years and above, we detected a noteworthy positive relationship between WWI and the incidence of moderate/severe periodontitis. This suggests a potential link between WWI and an escalated prevalence of moderate/severe periodontitis. Furthermore, when stratifying subgroups based on sex, education level, smoking status, and diabetes, the association between exposure variables and outcomes remained steadfast, indicating a consistent positive correlation. However, age stratification unveiled discrepant patterns as WWI demonstrated a negative association with the likelihood of moderate/severe periodontitis in the elderly age group (≥65 years) and a positive association in the younger and middle-aged groups (≥30 and <65 years).

To our knowledge, it is the first investigation to assess the link between WWI and periodontitis. According to a case-control study, the association between body weight and waist circumference with periodontitis varied with age. The risk of chronic periodontitis did not increase in adolescents aged 13 to 16. Still, it grew in adolescents aged 17 to 21 by 1.06 (adjusted odds ratio, 1.06 [95% CI, 1.01–1.09]) with each additional 1 kg in body weight and by 1.05 (adjusted odds ratio, 1.05 [95% CI, 1.01–1.08]) for every 1 cm rise in waist circumference [23]. In a longitudinal study by Gorman et al., which followed 893 non-diabetic men for 40 years, overweight men with high weight gain had more periodontal probing depth (PPD) events (PPD > 3 mm) compared to men with low weight gain. Overweight males with waist circumference increases of >0.14–0.39 cm/year or >0.39 cm/year encountered more PPD events compared to men with low growth [24]. Satpathy et al. also found that central adiposity and periodontal conditions uniquely raised the levels of serum interleukin-1β, with markedly elevated levels in individuals with central adiposity and periodontal disease, even after excluding diabetes and smoking [25]. An analysis of the relationship between obesity and periodontitis in individuals aged 13–34 encompassed 17 studies. 12 studies revealed a favorable association between obesity and periodontitis, with odds ratios varying from 1.1 to 4.5 for inflammation or periodontal destruction [26].

This research observed a possible link between WWI and a higher risk of moderate/severe periodontitis, with variations in different age groups. The increase in WWI may reflect the dysfunction of adipose tissue, leading to the synthesis and secretion of various pro-inflammatory cytokines [27]. Obesity may contribute to periodontitis through different pathological mechanisms, including changes in adipokine release levels, induction of inflammatory reactions, immune dysregulation, and dysbiosis of oral microbiota. Research in the past 20 years has confirmed the involvement of adipose tissue in regulating inflammation and immune responses related to the dysregulation or modified secretion of various pro-inflammatory and anti-inflammatory factors, including leptin, adiponectin, cytokines, and chemokines [28]. Nokhbehsaim et al. found that leptin significantly downregulated the levels of signaling molecules, such as TGF-β, VEGFA, and RUNX2, in human periodontal ligament cells, inhibiting the Smad/Mad-related signaling pathway and affecting the regenerative potential of human periodontal ligament cells [29]. In contrast, a study showed that transplantation of mesenchymal stem cells overexpressing leptin facilitated regeneration of periodontal tissues in a rodent model of osteoporosis [30]. The inconsistencies in research findings may be attributed to the heterogeneity between different studies. Studies have shown that serum concentrations of IL-1β, IL-6, TNF-α, and additional pro-inflammatory cytokines increase in obese mice, along with a significant increase in the ratio of nuclear factor-κB receptor activator ligand/osteoprotegerin in periodontal tissues [31]. Plasma levels of free fatty acids are often higher in obese patients, and these fatty acids can competitively bind to Toll-like receptor (Toll-like receptor, TLR) 2/4, inhibiting host immune defense against pathogens and leading to immune tolerance and dysbiosis of the periodontal microenvironment [32]. Maciel et al. found that periodontitis patients with obesity had a higher proportion of suspected periodontopathogens in subgingival biofilm, particularly Fusobacterium nucleatum, which increased with a higher body mass index [33]. A meta-analysis demonstrated a correlation between periodontitis and decreased Elevated high-density lipoprotein (HDL) levels and increased concentrations of low-density lipoprotein (LDL) and triglycerides [34]. Additionally, a study involving 162 Japanese participants revealed that periodontal infection stimulated lipolysis, resulting in elevated levels of circulating triglycerides [35]. Reduced high-density lipoprotein levels and heightened triglyceride and low-density lipoprotein levels contribute to an intense pro-inflammatory condition [36, 37]. Lipids can interfere with receptor and enzyme systems involved in cell membrane binding, further promoting the development of periodontal disease [38]. Aging may increase susceptibility to periodontitis due to changes in age-related innate immune and inflammatory states. Age-related intrinsic defects in innate immune cells in older individuals may make them more prone to immune and inflammatory dysregulation [39]. Aging is also associated with abdominal obesity and calorie intake [40]. In the elderly population, a significant positive correlation between WWI and osteoporosis has been found [41]. A 73-year study suggests that maintaining normal weight in youth and being slightly overweight in old age is associated with lower mortality rates [42]. A study conducted by Kotronia E. et al. in elderly individuals found a correlation between self-reported oral health, aggregation of oral health issues, and suboptimal dietary quality. Periodontal disease demonstrated an association with the proportion of calorie intake derived from saturated fats(OR = 1.48, 95%CI 1.09–2.01) [43]. The development of periodontitis is a complex multifactorial process. This study observed a negative correlation between WWI increase and moderate/severe periodontitis in the older age group, suggesting that the pathophysiological mechanisms in the geriatric population may be more specific and complex. The impact of obesity on this population is still controversial, and additional research is necessary to explore the association between WWI and periodontitis in this population and to examine the specific mechanisms between WWI and periodontitis in older people.

This research has several merits. Firstly, it is derived from NHANES data, a nationally representative large sample of population-based survey data obtained using a standardized protocol from 2009 to 2014, allowing for subgroup analysis. These strengths increase the credibility and applicability of the study. The authors also adjusted for confounding covariates, further improving the reliability of the results. As mentioned earlier, previous studies have investigated the examined the link between obesity and periodontitis. However, evaluating the association between WWI and periodontitis as an easily obtainable indicator may have more innovative and clinically practical significance for the prevention and early control of periodontitis. However, the limitations of this research cannot be ignored. As a cross-sectional study, it is important to note that due to potential temporal bias, it can only reveal patterns of association and not determine causation. Periodontal disease and obesity are associated with nutritional intake, and there is evidence that low dietary fiber intake is associated with obesity [44]. Similarly, studies have found an inverse relationship between dietary fiber intake and periodontal disease among U.S. adults aged 30 years and older [45], with patients with periodontitis having lower intakes of green and yellow vegetables and reduced intakes of hard foods [46]. This implies a possible reverse causal relationship. Secondly, although adjustments were made for some potential confounders, the authors can’t eliminate the impact of other potential confounding factors, such as dietary patterns (low calcium diet, high carbohydrate intake), genetic factors, and medication use. Statistical significance is due to large sample sizes with odds ratio values close to 1, as p-values are strongly influenced by sample size, and relying on the p-value alone may be limiting. In addition, unexplored confounding factors may also influence the observed association. Therefore, further large-scale cohort studies are essential to confirm the current results. Currently, evidence from a few intervention studies is limited, and using different periodontal indicators or criteria for measuring the presence and severity of periodontitis may lead to heterogeneity among studies. However, the global burden of periodontitis and the prevalence of obesity should still be given significant clinical and public health considerations.

## Conclusion

This study illustrates that a high WWI is linked to a heightened risk of moderate/severe periodontitis, and this association varies across different age groups. The findings of this study underscore the significance of WWI in identifying individuals at risk for periodontitis and have significant implications for the development of preventive and intervention strategies. Based on this association, comprehensive interventions, such as improving oral hygiene, controlling weight, and promoting healthy eating habits and lifestyles, may simultaneously reduce the occurrence of periodontitis and obesity and improve overall health. However, additional large-scale are still required to confirm our results.
